# Revisiting the Amyloid Cascade Hypothesis: From Anti-Aβ Therapeutics to Auspicious New Ways for Alzheimer’s Disease

**DOI:** 10.3390/ijms21165858

**Published:** 2020-08-14

**Authors:** Md. Sahab Uddin, Md. Tanvir Kabir, Md. Sohanur Rahman, Tapan Behl, Philippe Jeandet, Ghulam Md Ashraf, Agnieszka Najda, May N. Bin-Jumah, Hesham R. El-Seedi, Mohamed M. Abdel-Daim

**Affiliations:** 1 Department of Pharmacy, Southeast University, Dhaka 1213, Bangladesh; 2 Pharmakon Neuroscience Research Network, Dhaka 1207, Bangladesh; 3 Department of Pharmacy, BRAC University, Dhaka 1212, Bangladesh; tanvir_kbr@yahoo.com; 4 Department of Biochemistry and Molecular Biology, University of Rajshahi, Rajshahi 6205, Bangladesh; sohanbmb.ru@gmail.com; 5 Chitkara College of Pharmacy, Chitkara University, Punjab 140401, India; tapanbehl31@gmail.com; 6 Research Unit, Induced Resistance and Plant Bioprotection, EA 4707, SFR Condorcet FR CNRS 3417, Faculty of Sciences, University of Reims Champagne-Ardenne, PO Box 1039, 51687 Reims CEDEX 2, France; philippe.jeandet@univ-reims.fr; 7 King Fahd Medical Research Center, King Abdulaziz University, Jeddah 21589, Saudi Arabia; ashraf.gm@gmail.com; 8 Department of Medical Laboratory Technology, Faculty of Applied Medical Sciences, King Abdulaziz University, Jeddah 21589, Saudi Arabia; 9 Laboratory of Quality of Vegetables and Medicinal Plants, Department of Vegetable Crops and Medicinal Plants, University of Life Sciences in Lublin, 15 Akademicka Street, 20-950 Lublin, Poland; agnieszka.najda@up.lublin.pl; 10 Department of Biology, College of Science, Princess Nourah bint Abdulrahman University, Riyadh 11474, Saudi Arabia; may_binjumah@outlook.com; 11 International Research Center for Food Nutrition and Safety, Jiangsu University, Zhenjiang 212013, China; hesham.el-seedi@ilk.uu.se; 12 Pharmacognosy Group, Department of Pharmaceutical Biosciences, Uppsala University, SE-751 23 Uppsala, Sweden; 13 Department of Chemistry, Faculty of Science, Menoufia University, Shebin El-Koom 32512, Egypt; 14 Department of Zoology, College of Science, King Saud University, P.O. Box 2455, Riyadh 11451, Saudi Arabia; abdeldaim.m@vet.suez.edu.eg; 15 Pharmacology Department, Faculty of Veterinary Medicine, Suez Canal University, Ismailia 41522, Egypt

**Keywords:** Aβ, tau, Alzheimer’s disease, amyloid precursor protein, aducanumab, BAN2401

## Abstract

Alzheimer’s disease (AD) is the most prevalent neurodegenerative disorder related to age, characterized by the cerebral deposition of fibrils, which are made from the amyloid-β (Aβ), a peptide of 40–42 amino acids. The conversion of Aβ into neurotoxic oligomeric, fibrillar, and protofibrillar assemblies is supposed to be the main pathological event in AD. After Aβ accumulation, the clinical symptoms fall out predominantly due to the deficient brain clearance of the peptide. For several years, researchers have attempted to decline the Aβ monomer, oligomer, and aggregate levels, as well as plaques, employing agents that facilitate the reduction of Aβ and antagonize Aβ aggregation, or raise Aβ clearance from brain. Unluckily, broad clinical trials with mild to moderate AD participants have shown that these approaches were unsuccessful. Several clinical trials are running involving patients whose disease is at an early stage, but the preliminary outcomes are not clinically impressive. Many studies have been conducted against oligomers of Aβ which are the utmost neurotoxic molecular species. Trials with monoclonal antibodies directed against Aβ oligomers have exhibited exciting findings. Nevertheless, Aβ oligomers maintain equivalent states in both monomeric and aggregation forms; so, previously administered drugs that precisely decrease Aβ monomer or Aβ plaques ought to have displayed valuable clinical benefits. In this article, Aβ-based therapeutic strategies are discussed and several promising new ways to fight against AD are appraised.

## 1. Introduction

Alzheimer’s disease (AD) is the most common neurodegenerative disease, and globally, over 46 million people are affected by this devastating disease [1,2]. AD causes irreversible mental and cognitive deficiency including memory loss, personality disorder, and intellectual abnormality in patients older than 65 years [3,4]. Central sensory systems including the visual system are also affected during the advanced stages of the disease [5]. Collectively, complications of AD diminish the lifespan, hamper the quality of life, and cause physical impairment [6], which finally appears as a terrible difficulty in normal life activities [7]. To decrease the social and economic costs and the burden of the disease on patients and their families, recently, several attempts have been made to identify disease diagnostic markers [8]. Neuroimaging methods, such as magnetic resonance imaging and positron emission tomography, have been developed enabling researchers to diagnose AD at the early stages of the disease. Moreover, distinct biomarkers, which are essential to figure out the pathological characteristics of AD, have been observed in the cerebrospinal fluid (CSF) [9,10]. The advancement of AD is associated with three cardinal neuropathological features such as extracellular deposition of amyloid-β (Aβ) to produce neuritic plaques, intracellular neurofibrillary tangles (NFTs) and synaptic degeneration [11,12,13]. These pathological alterations arise in the neocortex, hippocampus, and other subcortical regions that are crucial for cognitive functions [14].

AD has been diagnosed on the basis of medical history, mental status tests, clinical findings, and brain imaging. Indeed, AD can exactly be identified only after death via relating clinical measures with an investigation of brain tissues upon autopsy. Currently, available therapies are mainly symptomatic and ineffective against the development of the disease [15]. Now, cholinesterase inhibitors [16] and the *N*-methyl-d-aspartate (NMDA) receptor antagonist memantine [17] merely represent the receivable options. Regardless of the enormous number of studies in AD pathogenesis, varieties of drug candidates entered into clinical trials, but no novel drug has been approved since memantine in 2003 [17]. Several arguments have been proposed to illustrate this failing, including an irrational selection of patients, various disease progression rates, minimal dosing, drug exposure, target engagement, improper time of intervention, inaccurate result measurements, and less effectiveness of clinical scales. Besides, imperfect perception regarding AD pathophysiology may facilitate the choice of vague targets.

Aβ has been considered as the foremost risk factor that playing a vital role in the initiation and progression of AD [18,19,20,21]. Aβ is generated into the typical individual, but in some instances, this peptide leads to aggregation, the starting point of disease progression. Many findings elucidate that Aβ oligomers might play a central role in neuronal dysfunction and AD [22,23]. Bennett et al. [24] reported that Aβ can increase tau pathology via elevating the generation of tau species that have the capacity to seed new aggregates. It was also observed that heterotypic seeded tau via pre-aggregated Aβ provides efficient seeds for prion-like initiation and propagation of tau pathology in vivo [25]. In tau-transgenic mouse models, NFTs formation was found to be increased due to the intrahippocampal infusion of synthetic Aβ fibrils, Aβ-rich extracts or pre-aggregated Aβ [25,26,27], which are similar to the activities observed in double-transgenic mouse models presenting both tau and Aβ pathology [28,29]. Human derived-tau injections in mouse models with a great Aβ plaque load boosted tau pathology, which is further indicating that Aβ plaques may induce tau propagation [30]. In this article, we have critically reviewed the recent studies targeting Aβ in Alzheimer’s pathogenesis.

## 2. Biomarkers for Alzheimer’s Disease

Decreased Aβ_1-42_ levels in the CSF can take place due to decreased Aβ clearance from the brain to the blood/CSF, along with increased deposition and aggregation of plaques in the brain. Alterations in Aβ levels in CSF vary in relation to the nature of the disease [31,32,33]. For instance, reduced levels of Aβ_1-37_ correlate with Lewy body dementia and Aβ_1-38_ levels with frontotemporal lobar degeneration [34]. In case of AD, levels of shorter Aβ_1-40_ forms remain unaffected or increased. Thus, evaluating the ratio of Aβ_1-42_/Aβ_1-40_ can improve AD diagnosis, but others have not observed such alterations [35,36]. Novel identification processes allow the measurement of Aβ oligomers, which may ameliorate the specificity of the diagnostic. For example, surface-enhanced laser desorption/ionization-time-of-flight-mass spectrometry has appeared as a valuable approach for the simultaneous identification and quantitation of various products generated through Aβ cleavage [37].

NFTs are another AD hallmark composed of highly phosphorylated forms of the microtubule-associated proteins tau [38,39]. Total tau levels in CSF elevate with age [40] such as < 300 pg/mL (i.e., 21–50 years), < 450 pg/mL (i.e., 51–70 years), and < 500 pg/mL (i.e., > 70 years) in healthy controls. Levels of total tau are considerably increased in AD individuals in comparison with the age-matched control participants with a cut off of > 600 pg/mL. Levels of total tau are also significantly increased in Creutzfeldt–Jakob disease (>3000 pg/mL). Levels of tau may also be a prognostic marker with a decent predictive validity for conversion from mild cognitive impairment (MCI) to AD since a high level of tau in CSF has been observed in 90% of MCI cases that later advance to AD, but not in stable MCI cases [41]. Study of other phosphorylated tau forms (i.e., phospho-tau-199, -231, -235, -396 and -404) may also provide substantial enhancements in early AD diagnosis [42].

Mo et al. [43] revealed that CSF levels of Aβ_1-42_ were decreased in AD as compared to non-AD dementia or controls. Nevertheless, the sole determination of the CSF levels of Aβ_1-42_ is not sufficient for reliable differential AD diagnosis. More studies are required based on the usage of biomarker combinations, such as Aβ_1-42_ levels in combination with other markers (e.g., tau, phosphorylated tau, Aβ_1-40_, total Aβ) to develop CSF biochemical measurements allowing reliable diagnosis of AD versus other non-AD cognitive deficits. In fact, total tau and phosphorylated tau levels rise in the CSF of AD subjects in comparison with the aged-matched subjects having normal cognition [44]. Positron-emission tomography (PET) studies revealed that accumulation of tau follows Aβ deposition in young individuals with autosomal dominant AD. In their study, Quiroz et al. [45] revealed that increased levels of tau were observed in medial temporal lobe areas in carriers of unimpaired PSEN1 E280A mutation in their late 30s, and that noticeable formation of tau tangle in neocortical areas was seen in one cognitively unaffected carrier as well as in individuals with mild cognitive impairment. In addition, these results also indicated that PET imaging of tau might be beneficial as a biomarker to differentiate people at high risk to develop clinical AD symptoms and to monitor the progression of the disease [45].

Aβ levels of blood plasma are elevated in Down syndrome and familial AD (FAD), though findings are inconsistent with sporadic AD [34]. It has also been revealed that concentrations of Aβ_1-40_ and Aβ_1-42_ can be decreased, increased or even unaltered in AD as compared to control individuals [31,34]. There was a noticeable rise in the levels of Aβ_1-42_ in plasma in females with MCI, but not in males, in comparison with age-matched, cognitively normal subjects [46]. It has been found by longitudinal studies that high concentrations of Aβ_1-42_ are also a risk factor for the development of AD, being this factor however not specific and sensitive for early AD diagnosis [47]. In AD, reduced levels of serum Aβ_1-42_ autoantibodies have also been observed [48], though no correlation has still been observed between Aβ levels in plasma and CSF. Antigen-spotted microarrays might be useful to validate and detect AD-selective biomarker autoantibodies in CSF and blood. Such a rapid method has also been developed for detecting differences between dissociated sera and the corresponding non-dissociated sera [49].

## 3. Amyloid Cascade Hypothesis

The amyloid precursor protein (APP) is a type I transmembrane glycoprotein containing 695–770 amino acids [50]. It is regarded that abnormal proteolytic processing of APP leads to the generation of Aβ [51]. In the non-amyloidogenic pathways, APP is cleaved by α- and γ-secretases. It is known that α-secretase is a metalloprotease (ADAM) which is localized to the Golgi complex and the cell membrane. This α-secretase can cleave APP at residue L688, located in the middle of the Aβ domain [52]. α-secretase causes APP cleavage leading to the formation of soluble APP alpha (sAPPα) and a cell-membrane-bound C-terminal fragment 83 (CTF83). The generated CTF83 is cleaved by γ-secretase to produce AICD and a small p3 fragment.

In the amyloidogenic pathways, APP is cleaved by β- and γ-secretases [50]. β-secretase (BACE1) belongs to the aspartic protease family and is around 500 residues in length containing 2 active sites situated at the lumenal side of the membrane [52]. The β-secretase enzyme possesses high sequence specificity [53,54], a feature that matches with BACE1, which can cleave APP at the *N*-terminal of the Aβ domain, either at residues Glu682 or Asp672 [52]. BACE1 also causes APP cleavage and generates soluble APP β (sAPPβ) and a cell-membrane-bound C-terminal fragment 99 (CTF99).

**Figure 1:**
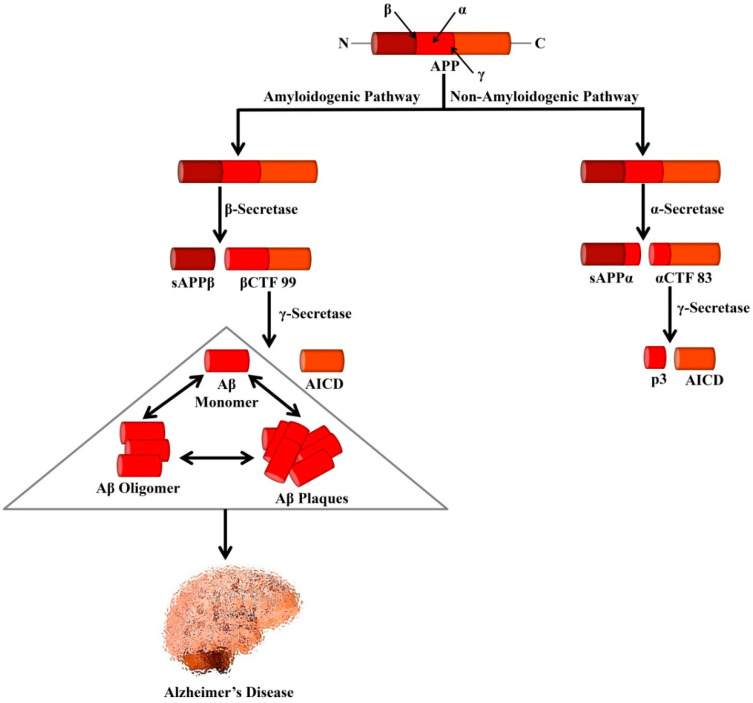
Processing of APP by secretases that leads to the formation of Alzheimer’s-associated Aβ peptides. In the amyloidogenic pathway, APP is cleaved by β- and γ-secretases leading to the formation of Aβ peptides and AICD. Formation of the neurotoxic Aβ is the major cause of AD. In the non-amyloidogenic pathway, APP is cleaved by α- and γ-secretases leading to the genesis of p3 and AICD. APP, Amyloid precursor protein; sAPPα, Soluble APP alpha; sAPPβ, Soluble APP beta; αCTF 83, Alpha C-terminal fragment 83; βCTF 99, Beta C-terminal fragment 99; AICD, APP intracellular domain.

On the other hand, γ-secretase is an enzymatic complex of four proteins including presenilin enhancer 2, presenilin (PSEN), anterior pharynx defective 1, and nicastrin. Indeed, each of the subunits is considered as an effective therapeutic for elevating Aβ clearance or controlling Aβ generation. Within the membrane, γ-secretase causes cleavage of βCTF99 that leads to the formation of the APP intracellular domain (AICD) and Aβ peptides. Various isoforms of Aβ isoforms are available that contain a variable length of amino acid residues including Aβ_1–40_ (i.e., the most abundant) and Aβ_1–42_ (i.e., the less soluble isoform). Aggregation of Aβ triggers the generation of protofibrils, fibrils, and oligomers, which eventually can lead to the formation of plaques, which are well-known as one of the major hallmarks of AD pathology as shown in Figure 1.

It is believed that in the AD process, Aβ accumulation in the brain is one of the events that take place initially [55]. Accumulation of Aβ also initiates in the entorhinal cortex and hippocampus. Furthermore, in NFTs, intracellular hyperphosphorylated tau deposition can cause disruption in axonal transport and progressive alterations in the cytoskeleton. In 1991, several independent groups initially proposed the theory that Aβ accumulation is the major characteristic of AD pathogenesis [56,57,58]. After one year, Hardy and Higgins [59] formally proposed the ‘amyloid cascade hypothesis’. As per the hypothesis, the deposition of Aβ in the brain can trigger several events including cognitive deficit, neuronal death, synaptic loss, formation of NFTs, and phosphorylation of tau.

The amyloid cascade hypothesis was further strengthened by the fact that AD may take place due to the autosomal dominant mutations in the *APP* gene and these mutations in *PSEN1* and *PSEN2* can elevate the Aβ generation and ultimately mediate the generation of Aβ aggregates and deposits [60]. Transgenic mouse models that express forms of PSEN proteins or APP containing mutations linked with human FAD progressively show the development of memory impairments and Aβ plaques in the brain, which further strengthens the hypothesis that buildup of Aβ can trigger AD [61]. Mutations in *PSEN* seem to be the major cause of FAD with over 150 causative mutations that have been mapped to the genes (*PSEN1* and *PSEN2*) encoding the PSEN proteins [62,63]. Most of these mutations seem to elevate the generation of Aβ_1–42_ over Aβ_1–40_ by mediating cleavage at residue 639 of APP over residue 637 [64]. Increased activity of BACE1 [65] and defective clearance [66] are found to contribute to the buildup of Aβ in the brain in late-onset sporadic AD (SAD). The buildup of Aβ is also found to be associated with the apolipoprotein E ε4 (*APOE ε4*) allele, which is regarded as the strongest genetic risk factor for late-onset SAD.

It has been revealed by post-mortem analyses that healthy elderly individuals can possess extensive amyloid pathology [67]. Moreover, brain imaging analyses showed that Aβ pathology was observed in up to 44% of cognitively healthy older people [68]. As compared to the individuals without amyloid pathology, a faster deficit in brain glucose metabolism, brain volume, and cognitive performance was experienced by individuals with amyloid deposits at baseline [69,70,71,72,73]. By 15–20 years, it has been estimated that the deposition of Aβ will precede the clinical AD symptoms [70].

## 4. Proteolytic Fragments of Amyloid Precursor Protein—CTF99 and sAPPα

Various studies have revealed that CTF99 generally accumulates within lysosomal, endosomal, and autophagic structures [74,75], which also correspond to the main intracellular sites for amyloidogenic APP processing [76]. In the 3xTgAD mouse model, accumulation of CTF99 did not take place due to elevated β-secretase or decreased γ-secretase cleavages, but rather because of an early lysosomal deficit [74]. Accumulation of CTF99 was also found to be similar in 3xTgAD and 2xTgAD (APPswe, TauP301L) mouse models, though the latter showed little if any, Aβ as estimated from the absence of mutated *PSEN1* [74]. In line with the lysosomal dysfunction in these two mouse strains, CTF99 accumulated within aberrantly large lamp- and cathepsins-positive structures, which number being also raised in CTF99-positive neurons [74]. Findings from mouse models revealed that the accumulation of CTF99 may play a role in this pathology. Pharmacological suppression of γ-secretase in young animal models not only resulted in elevated levels of CTF99 but also worsened lysosomal dysfunction [75].

In CTF99-expressing mouse models, a study has studied the hippocampal long-term potentiation (LTP) to analyze the effect of CTF99 in synaptic alterations [75]. Interestingly, as compared to control mice infected with control virus, hippocampal LTP was found to be considerably decreased in young CTF99-expressing mouse models. Suppression of γ-secretase did not rescue LTP alterations, which is suggesting that CTF99 instead of Aβ, induced these activities [75].

Indeed, sAPPα shows a remarkable neuroprotective effect. Numerous activities of sAPPα have been highlighted through in vitro studies. Long-term survival of cultured cortical neurons can be enhanced by sAPPα and it is assumed that it has a significant contribution in protecting cultured neuroblastoma cells against glutamate toxicity [77], since it can protect cultured neuronal cells against metabolic, excitotoxic, and oxidative damages [78,79]. Findings of in vivo studies are in line with those of in vitro studies. In addition, sAPPα can also induce cortical synaptogenesis and neurite outgrowth [78,79,80]. In the subventricular zone of the lateral ventricle in adult mouse models, sAPPα functions together with epidermal growth factors to play a role as a growth factor for neuronal progenitor cells [81], which is further indicating the activity of sAPPα in adult neurogenesis as these cells hold the ability to generate new neurons during adulthood.

In a transgenic AD mouse model and cell culture, BACE1 modulation through sAPPα led to a decreased level of Aβ generation, and plaques [82]. Furthermore, it was also demonstrated that sAPPα can play a role as an endogenous inhibitor of BACE1. It was also confirmed that sAPPα reduces the BACE1 activity via binding with its allosteric site [83]. Moreover, sAPPα suppressed the activity of the glycogen synthase kinase 3beta (GSK3β) and BACE1 by acting through unknown receptors, which eventually resulted in decreased tau phosphorylation [84].

## 5. Crosstalk of Aβ and Tau

Molecular, genetic, and neuropathological data indicate that AD pathology can be mediated by the tau protein. Pathology of tau is associated with AD severity and duration [85,86,87] and also with the neuronal loss [88,89]. Furthermore, tau pathology also facilitates the association between the occurrence of AD and load of brain Aβ [86], which is evident in the entorhinal cortex in individuals with subjective memory complaints [90]. Without the presence of Aβ, deposition of tau in the hippocampus may be ineffective in inducing the neurodegenerative mechanisms which can cause AD [91]. In individuals with SAD [92] or FAD [93], longitudinal analyses have revealed that the levels of tau in CSF increase in the early stages of the disease but fall once the symptoms appear. Outcomes of a study involving stable isotope labeling kinetics analysis concluded that the pathology of Aβ can increase tau production [94]. However, these results contradict the hypothesis that increased CSF levels of tau in individuals with AD can take place initially from dying and dead neurons. A certain type of tau aggregate formation can be induced by the neuritic Aβ plaques, which can lead to the generation and distribution of neuropil threads and NFTs [30]. Increased tau levels and decreased Aβ_1–42_ levels are linked with the development of AD in individuals with MCI [95].

Post-mortem examinations based on confirmed AD cases have revealed what are the main cell types and functional systems influenced by the disease, though the primary sites of the pathology remain still not clear. As per the modified and original Braak staging protocols [96,97], primary sites of tau pathology are located within the transentorhinal and entorhinal cortex (stage I), propagated into the hippocampus (stage II), temporal cortex (stage III) and ultimately into other cerebral cortex areas (stage IV), eventually reaching the visual association cortex region (stage V) and primary visual cortex area (stage VI). In contrast, the order of Aβ plaque deposition [98] initiates within neocortical areas, especially within the temporal lobe region (phase 1), and reaches allocortical areas including amygdala and hippocampus (phase 2), and after that into subcortical areas (phase 3), brain stem (phase 4) and eventually cerebellum (phase 5) [99].

Tau interacts through its amino-terminal projection domain with the Fyn kinase (Fynomers) [100]. Fyn can phosphorylate the NMDA receptor subunit 2 to mediate the interaction of the NR complex with the postsynaptic density protein 95 (PSD-95) [101,102,103], connecting NRs to synaptic excitotoxic downstream signaling [104]. Without affecting synaptic NMDA currents, disturbance of the NR/PSD-95 interaction averted excitotoxic injury in a rat model of stroke and cultured neurons [105]. In APP transgenic mouse models, Fyn reduction prevented Aβ toxicity, whereas its overexpression enhanced toxicity [106,107].

In their study, Ittner et al. [12] generated transgenic mice (Δtau74) that only express the amino-terminal projection domain of tau and crossed them with Aβ-forming APP23 and tau^−/−^ mice to identify how tau can cause Aβ toxicity. It was observed that tau possesses a dendritic activity in postsynaptic targeting of the Src kinase Fyn, a substrate of which is the NMDA receptor. Tau missorting in transgenic mouse models expressing truncated tau (Δtau) and absence of tau in tau^−/−^ mouse models both disrupted postsynaptic Fyn targeting, which further uncoupled NMDA receptor-induced excitotoxicity and henceforth mitigated Aβ toxicity. Δtau expression as well as tau deficiency prevent memory impairments and ameliorates survival in Aβ-forming APP23 mouse models. Furthermore, these impairments were also completely rescued with a peptide that uncouples the Fyn-induced in vivo interaction of PSD-95 and NMDA receptors. These results indicate that the dendritic activity of tau confers Aβ toxicity at the postsynapse level with direct involvement in AD pathogenesis and treatment [12].

Kim et al. [108] have confirmed that CTFs such as C31 and AICD [C57, C59] induce neurotoxicity on rat primary cortical neurons and differentiated PC12 cells via the stimulation of the expression of GSK3β, generating a ternary complex with CP2/LSF/LBP1 and Fe65 in the nucleus, while a point mutant and deletion mutants with Y682G of the YENPTY domain, a Fe65 binding domain, do not. Besides, expression of APP770 and the Swedish mutant form of APP elevated CTFs in neuronal cells and also stimulated GSK3β up-regulation at both the protein and mRNA levels. Furthermore, the CP2/LSF/LBP1 binding site in human GSK3β promoter region is important for the stimulation of gene transcription by CTFs. The neurotoxicity mediated by CTFs (i.e., AICD and C31) was accompanied by a rise in the active form of GSK3β, and trough the stimulation of tau phosphorylation as well as a decrease in the levels of nuclear beta-catenin, resulting in apoptosis [108].

## 6. Therapeutic Targeting of Aβ in Alzheimer’s Pathogenesis

### 6.1. Decreasing Aβ Production

#### 6.1.1. α-Secretase Activators

Since α-secretase processing of APP includes cleavage within the Aβ peptide sequence, preventing the formation of Aβ, activation of APP α-secretase cleavage is regarded as an effective AD treatment [109]. Multiple drugs are currently in use for AD treatment that can elevate the effect of α-secretases by activating related signaling cascades and this has been regarded as the best therapeutic technique, so far [110,111,112]. On the other hand, selegiline (i.e., a selective inhibitor of monoamine oxidase) was utilized to slow the progression of AD, which has been found to elevate the activity of α-secretase through a protein trafficking-related process [113,114]. Following the observation that the chronic use of statin might be protective, atorvastatin was employed for AD treatment, inducing α-secretase activation [115,116]. Feldman et al. [117] reported in a large-scale randomized controlled trial of 640 mild to moderate AD patients, that atorvastatin was not associated with a significant clinical benefit over 72 weeks.

Numerous drugs anticipated as indirect activators of α-secretase have moved to the clinical trial stage for AD. Among these drugs, etazolate (EHT 0202, a modulator of GABA receptor) was found to be the most prominent one and has reached phase II clinical trials based on the observation that it stimulated sAPPα generation and provided protection against Aβ mediated toxicity in rat cortical neurons [118,119]. Later EHT 0202 failed to show any significant cognitive or clinical benefit in an over 3-month, double-blind, placebo-controlled study in 159 mild to moderate AD patients [118]. It was reported in 2008 that PRX-03140 (i.e., an agonist of 5-HT4) can induce α-secretase and may exhibit positive outcomes in phase II clinical trial (NCT00693004) with an enhancement of the cognitive functions in AD individuals, though these experiments were not followed by additional studies [120]. Epigallocatechin-gallate (EGCG), which is a polyphenolic compound extracted from green tea, induces α-secretase activity via the protein kinase C (PKC) signaling pathway and decreases cerebral amyloid deposition in AD mouse models [121,122,123]. The benefits of this compound in AD are being studied in phase II/III trials (NCT00951834) [123]. Bryostatin (i.e., a strong modulator of PKC) can elevate the release of the α-secretase product, sAPPα. However, in a phase II study, bryostatin failed to show any significant cognitive or clinical benefit in a 12-week, double-blind, placebo-controlled study in 150 advanced AD patients [124].

#### 6.1.2. β-Secretase Inhibitors

As stated earlier, the protease BACE1 is accountable for the primary cleavage of APP, which increases the production of Aβ [125,126]. The relationship between Aβ decline and BACE1 inhibition was reported in numerous studies with BACE1 knock-out mice [127,128]. Furthermore, it was observed that inhibition of BACE1 rescued Aβ-driven cholinergic dysfunction and improved memory deficits in APP transgenic mice [129,130].

Several BACE1 inhibitors containing properties including regulation of insulin metabolism are derived from approved drugs that are used in type 2 diabetes. Elevation of ubiquitination and inhibition of β-secretase to degrade the amyloid load can be achieved via peroxisome proliferator-activated receptor gamma (PPAR-γ) activation by thiazolidinediones [131]. Reduction of the peripheral insulin resistance can be achieved by PPARγ agonists, including thiazolidinedione derivatives (i.e., pioglitazone and rosiglitazone) [132], which can mediate aggravation of neuropathology of AD. This weakening of insulin sensitivity can help in proteolysis of Aβ. Nonetheless, pioglitazone failed to show cognitive or clinical benefits in a large prevention trial (NCT01931566) owing to the lack of efficacy of the drug and no safety concern [133]. On the other hand, in 2 double-blind, placebo-controlled trials, rosiglitazone failed to show any clinically significant efficacy in cognition or global function in mild to moderate AD patients for 48 weeks [134].

There is an ongoing investigation of several novel drugs. CTS-21166, which displays measurable brain penetration properties, lessened the plasma levels of Aβ and showed good tolerance in healthy individuals [135,136,137]. Findings from the phase I trial indicated that CTS-21166 is safe when injected intravenously in AD individuals at doses as high as 225 mg. Findings revealed a dose-dependent decrease of Aβ concentrations in plasma for a prolonged time [138]. A marked decrease in the Aβ levels in plasma lasted over 72 h [139]. Similar observations were also achieved from a second phase I clinical trial on participants receiving an oral liquid solution of 200 mg CTS-21166 [139]. Despite these initial encouraging results, CTS-21166 has not moved further in clinical trials and the 6-year collaboration between Astellas Pharma and CoMentis for developing and commercializing CTS-21166 was terminated in 2014 [140].

BI 1181181 (i.e., an orally active BACE1 inhibitor) is the first generation of BACE1 inhibitors that failed in phase I clinical trials due to their low blood-brain barrier penetration and low oral bioavailability. Subsequently, the second generation of BACE1 inhibitors including LY2886721, LY2811376, and RG7129 also failed in clinical studies owing to liver toxicity [141]. On the other hand, the third-generation of BACE1 inhibitors including JNJ-54861911, CNP520, and AZD3293 was discontinued because of the worsening of cognitive performances compared to placebo. Even though verubecestat (MK-8931) decreased Aβ levels in the central nervous system (CNS) of animal models and AD individuals [142] upon a 104-week, randomized, double-blind, placebo-controlled trial, this compound failed to improve clinical ratings of dementia among patients with prodromal AD [143].

#### 6.1.3. γ-Secretase Inhibitors

Like BACE1, Aβ generation is a result of APP cleavage, which is mediated by γ-secretase. Henceforth, γ-secretase is regarded as a principal target in AD therapy [144,145]. This enzyme complex is involved in various physiological processes [146]. In addition to APP, there are copious substrates in the human body that γ-secretase can react with; many of them are neuronal substrates [147]. On the other hand, cleavage of Notch 1 is also done by γ-secretase, which can ultimately lead to Notch intracellular domain release and can consequently translocate to the nucleus to cause gene regulation associated with cell survival, determination of cell fate, and cell development [148]. Henceforth, to avoid the drawbacks related to abnormalities of Notch-signaling, γ-secretase inhibitors should be carefully designed. Frequently observed adverse effects of γ-secretase inhibition include changes in hair color [149], skin reactions [150,151], gastrointestinal [152], and hematological [153] toxicity. Some inhibitors of the γ-secretase have been tested during clinical studies and most of them have been found to decrease Aβ production in CSF or plasma. Nonetheless, few of them effectively avoided the side-effects induced by Notch.

A decrease in the levels of Aβ in plasma and downregulation of Aβ production in the CNS have been achieved with semagacestat (LY450139), [154] a γ-secretase inhibitor which has been forwarded to phase III clinical trials. In phase I trials, a dose-dependent reduction in the synthesis of Aβ was observed in CSF [154]. Conversely, in phase II clinical trials, side effects related to the skin were observed. However, there was a significant decrease in the plasma Aβ level; a similar trend was not noticed in CSF and no momentous activities on function and cognition were reported. Two essential phase III clinical trials were hesitantly commenced, and subsequently discontinued as a result of a lack of efficacy, increased risk of the skin infection, and cancer [155]. Fall of semagacestat and recurrent unsatisfactory results with other γ-secretase inhibitors suggested the idea that an interaction between the four subunits of the γ-secretase and their substrates is needed.

While showing target specificity, different γ-secretase inhibitors have exhibited suitable interactions with the γ-secretase subunits. Sulfonamide and MRK-560 based γ-secretase inhibitors predominantly inhibit PSEN1 rather than PSEN2, whereas L-685458 and DAPT displayed the lowest selectivity [156,157]. Even though the possibility of drug design still appears challenging, heterogeneity of Aph1 is important for the survival of individuals, which further recommends that targeting the Aph1b γ-secretase particularly would be better tolerated [158]. Therefore, selective inhibition of specific sites via the next-generation inhibitors of Notch-sparing γ-secretase came into focus and NIC5-15, begacestat (GSI-953), and avagacestat (BMS-708163) represent such inhibitors under clinical study. Moreover, it was found that, avagacestat possesses 137 times more selectivity over Notch for APP in cell cultures. In rats and dogs, avagacestat strongly decreases the levels of Aβ in CSF without producing any Notch-related toxicity [159]. Avagacestat was primarily supposed to play a role as an inhibitor of Notch-sparing γ-secretase. Nonetheless, further experiments indicated this to be false [160]. In 2010, due to liver toxicity, a clinical trial for the Notch-sparing inhibitor ELND006 (Elan Corporation) was terminated [161]. Tarenflurbil (i.e., a γ-secretase modulator) exerted positive effects on cognitive functions in phase II clinical trial but was stopped following phase III clinical trials because of poor outcomes [162,163,164]. CHF-5074 (i.e., Chiesi’s γ-secretase modulator) reached phase II clinical trials, but experiments were terminated for unrevealed causes [165]. All drugs, including Notch-sparing compounds, were discontinued for lack of efficacy and poor tolerability.

A decrease in the concentration of Aβ in the plasma, but not in CSF was noticed with a second Notch-sparing inhibitor, begacestat [148,166,167]. A phase I clinical trial in association with donepezil has also been carried out but additional data regarding this trial are not available [167]. On the other hand, NIC5-15 or pinitol, a naturally-occurring cyclic sugar alcohol displays good safety and tolerance and is presently under a phase II clinical trial [168,169].

### 6.2. Blocking Aβ Aggregation

#### 6.2.1. Anti-Amyloid Aggregators

Tramiprosate is a small and orally-administered aminosulfonate compound that has the ability to bind with Lys28, Lys16, and Asp23 of Aβ42 [170]. Interestingly, this binding can lead to Aβ42 monomer stabilization, further decreasing fibrillar (plaque) and oligomeric amyloid aggregation. It was reported that suppression of oligomer generation and elongation can exert neuroprotective effects against Aβ-mediated subsequent deposition [171,172,173,174]. In a transgenic AD mouse model, it was seen that when cyclohexanehexol stereoisomers were administered orally, Aβ aggregation into high-molecular-weight oligomers was suppressed in the brain and improvement of various AD-like phenotypes in the mouse models were observed [175]. Based on good safety and tolerance profile, scyllo-inositol (i.e., a cyclohexanehexol stereoisomer) is being studied in phase II clinical trials with mild-to-moderate AD individuals [176]. On the other hand, EGCG was also found to induce the generation of nontoxic, unstructured Aβ aggregates by suppressing its structural alteration from a random-coil to a β-sheet structure [177]. The generated small Aβ oligomers in the presence of EGCG were harmless and unable to seed further fibril growth [178]. Curcumin which is a diphenol extracted from the rhizomes of *Curcuma longa* (turmeric), reduced the concentrations of soluble and insoluble Aβ and plaque burden in Tg2576 transgenic mouse models [179]. Curcumin can bind with both Aβ fibrils and oligomers [180]. Furthermore, it is believed that curcumin can also suppress the oligomerization of Aβ, mediate the nontoxic Aβ fibrils deposition, and reduce the neurotoxicity of Aβ aggregates [181,182]. Therefore, it might be sufficient enough to accelerate or change the pathway from toxic Aβ oligomers to nontoxic Aβ forms. Moreover, curcumin also induces the conversion of Aβ fibrils by decreasing the prefibrillar Aβ species and thus alleviates toxicity of Aβ in the transgenic *Drosophila* model [183]. However, curcumin in a 24-week, double-blind, placebo-controlled trial in mild to moderate AD patients failed to demonstrate any biological or clinical effects [184].

Resveratrol is a non-flavonoid polyphenol that is abundantly found in red wine and many plants [185]. Multiple studies have suggested that the neuroprotective activity of resveratrol on AD is credited to several factors including proteasome-mediated intracellular Aβ degradation [186], reduction in secreted and intracellular Aβ through inhibition of BACE1 [187], and reduction of Aβ-induced toxicity [188]. Furthermore, resveratrol can decrease the accumulation of extracellular Aβ by elevating the activity of the AMP-activated protein kinase (AMPK) to induce autophagy and lysosomal degradation of Aβ [189]. In a 52-week, double-blind, placebo-controlled study in 119 mild-to-moderate AD patients, the brain volume loss was increased by a resveratrol treatment compared to placebo [190]. The clinical effect trajectories of resveratrol remain uncertain in another 12-month study in mild-to-moderate AD [191]. Finally, flavonoids including quercetin and myricetin showed neuroprotective activities in AD via several mechanisms including inhibition of BACE1 activity [192], activation of AMPK [193], and antioxidative effects [194,195]. Quercetin has a strong capacity to suppress the formation of Aβ fibrils and protect neuronal cells from Aβ-mediated toxicity [196]. On the other hand, myricetin prevented conformational alteration of Aβ from a random-coil to a β-sheet-rich structure, and its suppressive activity against aggregation of Aβ was directly proportional to the number of hydroxyl groups on the B-ring [197].

#### 6.2.2. Metal Chelators

Aβ-metal adducts and metal ions can produce toxic radical species that have the ability to modify biomolecules, which can further lead to neuronal death [198]. Increased concentrations of redox-active metal ions such as copper (Cu)/iron (Fe) might lead to mitochondrial dysfunction, DNA damage, and reactive oxygen species (ROS) generation [199]. Furthermore, these metal ions can cause neuronal cell damages via mediating the generation of ROS or nitrogenated species (RNS) containing unpaired electrons, primarily characterized by peroxynitrite, nitric oxide, lipid peroxyl and alkoxyl radicals, hydroxyl, and superoxide anion radical [200,201,202]. In their study, House et al. [203] revealed that Aβ_1-42_ alone or in the presence of Fe(III) or Al(III) generated β-pleated sheets of plaque-like amyloids which were dissolved following incubation with either ethylenediaminetetraacetic acid (EDTA) or desferrioxamine (DFO). Cu(II) prevented while Zn(II) suppressed the generation of β-pleated sheets of Aβ_1-42_. However, neither of these effects was influenced by incubation of the aged peptide aggregates with either EDTA or DFO. Existence of marked levels of Fe(III) and Al(III) as contaminants of Aβ_1-42_ preparation indicated that these two metals were associated with either inducing the generation or stabilizing the structure of β-pleated amyloid [203]. Khan et al. [204] evaluated the effect of Aβ_1-42_ aggregation in mediating the redox cycling of Fe(II) and the associated activities of companion metals, zinc (Zn), Cu, and aluminum (Al). It was mentioned that the main effects of Aβ_1-42 in the redox cycling of iron were perhaps in binding Fe(III) and thus slowing its precipitation as redox-inactive Fe(OH)3_(s). Additional aggregation state-specific binding of both Fe(III) and Fe(II) determined critical equilibrium associated with H_2_O_2_ generation via the superoxide anion (O_2_^−^) in favor of keeping Fe(II) in solution. Introduction of physiologically important levels of either Zn(II) or Cu(II) decreased the activity of Aβ_1-42 in the Fe(II)/Fe(III) redox cycle, while a pathophysiologically important Al(III) level induced the redox cycle in favor of Fe(II) whether or not Zn(II) or Cu(II) were additionally present. Collectively, these findings suggest the idea that oxidative stress in the vicinity of senile plaques might take place due to Fenton chemistry catalyzed by the co-deposition of Aβ1-42_ with various metals including Al(III) and Fe(II)/Fe(III) [204].

Henceforth, metal chelators that can impede Aβ burden, can be envisaged as a therapeutic approach. Instead, clioquinol (i.e., metal-induced Aβ inhibitors) can increase Aβ degradation via the redistribution of metal ions to neurons thus stimulating expression of metalloproteinases [205]. Evidence of the decrease in the concentration of Aβ_1-42 in CSF and enhancement of behavioral and cognitive performances were noticed in phase II clinical trials of copper/zinc ionophore PBT2 [206]. Levels of Fe, Zn, and Cu are found to be high around and in amyloid plaques in AD brain [202,207,208,209,210]. Higher concentrations of Fe [211] and Zn [212] were also identified in the amyloid plaques of the Tg2576 (APPsw) mouse model for AD. Interestingly, Aβ shows high selectivity and low-affinity Cu_^2+^- and Zn^2+^-binding sites that induce its aggregation via interactions with Zn^2+^, Cu^2+^, and to a lesser extent with Fe^3+^ in vitro [213,214,215]. Nuclear magnetic resonance and electron paramagnetic resonance studies suggested a model of monomer Aβ binding to a Cu ion via 3 histidine and tyrosine residues or via a bridging histidine for aggregated Aβ [216]. Raman spectroscopy of senile plaque cores confirmed that Zn and Cu ions are coordinated through histidine residues [210], which are situated at the N-terminal end of the Aβ sequence. Indeed, the affinity of Aβ variants for Cu^2+^ is highest for Aβ_1-42_ > Aβ_1-40_ > > rat Aβ, in association with toxicity and redox activity [214,215,217].

A significant association between amyloid formation and Cu levels has been reported by 2 complementary and independent studies using 2 different transgenic APP mouse models. Both of these studies stated reduced constitutive brain Cu levels [218,219], which is in line with earlier results [220]. Increased levels of Cu in the brain, either by the introduction of a mutant allele of the CuATPase7b Cu transporter [219] or by dietary Cu supplementation [218], caused a marked decrease in Aβ and amyloid plaque load and ameliorated mice survival. These observations indicate that increased Cu levels might trigger non-amyloidogenic APP processing, as confirmed previously in vitro [221].

### 6.3. Increasing Aβ Clearance

#### 6.3.1. Aβ Vaccination

Aβ vaccination has been tested and found to be effective against cognitive impairment, cellular changes, and Aβ pathology in animal models of AD [222,223]. In PDAPP transgenic mice (i.e., which overexpress mutant human APP), most of the neuropathological AD hallmarks develop progressively in a brain-region- and age-dependent manner. It was noticed that a transgenic mouse following Aβ vaccination was found to overexpress a mutant form of the human APP, which is protected against the formation of amyloid plaque, neuritic dystrophy, and astrogliosis [224]. Besides, apart from protecting the aggregation of Aβ, it also helps in clearing the amyloids from the brains of adult mouse models [225].

##### Active Immunization

Active immunotherapies have been developed to activate an immune response in the human body of specific antibodies against Aβ [226]. Furthermore, active immunotherapies might be utilized to mediate anti-Aβ antibody responses in individuals with early stages of AD. Considering the potentially hazardous autoimmune responses because of newly activated Aβ_1-42_ specific inflammatory T cells, new peptide vaccines were developed in which the parts accountable for the activation of T cells were removed and only the parts required for Aβ production-specific antibodies persisted [227]. Currently, 3 of these peptide vaccines for active immunization including affitope, ACC001, and CAD106 are in phase II clinical trials [227]. In case of all 3 approaches, phase I trials exhibited positive antibody responses with no signs for adverse autoimmune inflammation and clinical trials of these active immunization therapies are being continued [227]. CAD106 has been developed to exert a powerful antibody response while escaping the activation of inflammatory T cells [228]. Interestingly, CAD106 can combine with multiple Aβ_1-6_ copies derived from the *N*-terminal B cell epitope of Aβ, coupled to a Qβ virus-like particle. CAD106 stimulated Aβ-antibody titers without activating the Aβ-reactive T cells in animal models. CAD106 administration resulted in a decrease of amyloid accumulation in the brain of APP-transgenic mouse models [229]. In patients with mild AD, CAD106 [230] has finished the phase II clinical trial and did not cause meningoencephalitis [231].

A different active immunization method was also established that mimics the *N*-terminus of Aβ. Affitope, AD01, and AD02 are keyhole limpet hemocyanin vaccines with a synthetic peptide of 6 amino acids that represent the natural Aβ sequence. In animal models, AD01 as well as AD02, mimic the *N*-terminus of Aβ [232]. Furthermore, the controlled selectivity averts cross-reactivity with APP, and the small size prevents autoreactive T-cell activation [233]. To examine tolerability and clinical/immunological activity, AD02 has been selected for development in phase II trial in early AD individuals [234]. It was found that AD02 could not meet its primary endpoints in early AD. IMM-AD04 (2 mg) vaccination alone (utilized in one of the 2 control arms) provided statistically significant slowing of global, functional, and cognitive decline as revealed by primary and secondary outcomes over 18 months [235]. ACC-001 is a conjugate of several short Aβ fragments associated with a carrier composed of inactivated diphtheria toxins. It was developed to avoid the safety issues linked with the prior AN1792 active vaccine against full-length Aβ_1-42_ [236]. However, ACC-001 was discontinued in phase II trials because of a strong autoimmune response [237,238,239].

##### Passive Immunization

Direct administration of antibodies is another approach to evade the immune response. Passive immunization is effective for the removal of amyloid plaques and rescuing glial and neuritic pathology [222]. Moreover, the decrease in early cytopathology [240] and hyperphosphorylation of tau [241], as well as the reversal of abnormal hippocampal synaptic plasticity [242] can also be achieved with the passive immunization. Rescue of synapse loss in brains of APP transgenic mouse models and Aβ plaques removal can be mediated by bapineuzumab (i.e., a humanized monoclonal antibody) [222]. In AD individuals, bapineuzumab was first used as passive immunotherapy. In 2 phase III clinical trials of bapineuzumab in mild-to-moderate AD individuals, Salloway et al. [243] stated that bapineuzumab did not ameliorate clinical outcomes in AD individuals, notwithstanding treatment differences in biomarkers seen in carriers of *APOE ε4*. Solanezumab is a humanized antibody as bapineuzumab. Nonetheless, solanezumab is targeted to an internal epitope of Aβ_13-28_. Furthermore, solanezumab exhibited superior binding to soluble Aβ rather than fibrillar Aβ. Early clinical trials exhibited minor enhancement in cognitive functions in individuals with moderate AD [244]. However, individuals with mild AD exhibited a 33% decrease in a rate of decline so a phase III clinical trial was started to analyze the treatment of mild AD individuals with solanezumab [245]. Moreover, early clinical trials revealed that solanezumab possesses similar efficacy in individuals without or with the *APOE4* allele [246] as compared to the bapineuzumab trial. Solanezumab was discontinued for the treatment of sporadic AD.

Gantenerumab (RG1450, RO4909832) is the only fully developed human antibody. The most probable mechanism of the effects of this antibody seems to be the binding to small plaques and stimulation of a phagocytic response by microglia [247]. In clinical trials, when gantenerumab was administered to moderate AD individuals, it decreased the load of brain amyloid by up to 30% as estimated via a positron emission tomography scan, but 2 individuals experienced vasogenic cerebral edema in the high dose group [248]. In a phase II/III clinical trial (NCT01760005), Hoffmann La-Roche is working together with Eli Lilly run by Washington University School of Medicine. A randomized clinical trial (DIAN-TU-001) aims to evaluate gantenerumab and Lilly’s solanezumab in dominantly inherited AD patients (a rare, early-onset type of AD) [249,250,251]. This clinical study is estimated to be finished in March 2021. Crenezumab (a humanized antibody) is used to target Aβ with a modified human immunoglobulin G (IgG) isotype IgG4 to decrease the affinity for Fc receptor binding and decrease the risk of immune cell stimulation permitting higher dosing of the antibody as compared to that allowed with previous immunotherapy methods. It has been observed that crenezumab is likely to bind with various forms of Aβ including fibrillar, oligomeric, and monomeric forms [252]. Phase I clinical trials revealed better safety data and currently, crenezumab is entering into phase II clinical trial. Unlike other tested antibodies, ponezumab (another humanized IgG antibody) targets Aβ_1-40_ without targeting the full-length APP protein [253,254]. Ponezumab is now being analyzed as a treatment for cerebral amyloid angiopathy [245].

Aducanumab (BIIB037) is a human monoclonal antibody (mAb) that has the capacity to bind with various aggregated forms of Aβ such as, insoluble fibrils and soluble oligomers. However, it has been reported that this mAb does not bind with Aβ monomers. In an ongoing PRIME study (NCT01677572, phase Ib trial), aducanumab was shown to reduce Aβ plaques and slow the decline in clinical measures in prodromal or mild AD patients, with acceptable tolerability and safety [255]. Nevertheless, a side effect called amyloid-related imaging abnormalities (ARIA) was also observed along with Aβ removal in a dose-dependent manner and was mainly reported in *APOE4* carriers as compared to the non-carriers. Based on the findings of PRIME and observations from previous clinical trials in AD individuals, current phase III trials of aducanumab in early AD individuals including EMERGE (NCT02484547) and ENGAGE (NCT02477800) have been designed [256,257]. Features of those study designs involve selection of participants with confirmed Aβ pathology, in order to confirm adequate target engagement, and carrying out clinical trials in participants at early symptomatic AD stages. It has been reported that the EMERGE study met its primary endpoint exhibiting a marked decrease in clinical decline in comparison with the placebo. Furthermore, Biogen (an American biotechnology company, Cambridge, MA, USA) also revealed findings from a subset of individuals in the ENGAGE study, who were randomized to the high dose of aducanumab (10 mg/kg), that is, similar with the EMERGE results [258]. As an AD treatment, Biogen declared the completion of the submission of Biologics License Application to the FDA for aducanumab on 8 July 2020. Following its approval, aducanumab will become the first therapeutic agent to decrease the AD-related clinical decline. In addition, this mAb will also become the first therapeutic agent to confirm that Aβ clearance can lead to better clinical outcomes [259].

BAN2401 is a humanized IgG1 version of the mouse monoclonal antibody mAb158 that has the capacity to bind with soluble and large Aβ protofibrils [260]. It was observed that mAB158 can decrease Aβ protofibrils in CSF and brain of Tg-ArcSwe mouse models [261]. Following experiments in mouse neuron-glial, co-cultures revealed that mAb158 might protect neurons (decrease toxicity of Aβ protofibrils) via resisting the pathological buildup of these protofibrils in astrocytes [262]. In March 2014, this mAb was licensed to Eisai and collaboration was established with Biogen to develop BAN2401. In a different phase III trial (NCT03887455), the efficacy of BAN2401 is being evaluated in early AD patients [263]. This phase III trial is projected to be completed in July 2024.

#### 6.3.2. Receptor-Mediated Clearance

The receptor for advanced lipoprotein receptor-related protein (LRP) and glycation end products (RAGE) are both considered as multi-ligand receptors binding with copious ligands [264], which mediate the transport of Aβ out of the brain [265,266]. LRP1 is found to bind to many structurally different ligands, such as APP, Aβ, lactoferrin, and ApoE [267]. A low level of LRP1 expression or LRP1 antagonists can increase the load of Aβ [268,269].

Soluble LRP can be produced due to the cleavage of the extracellular LRP domain by β-secretase. Besides, β-secretase can also bind to free Aβ in the plasma to decrease the extracellular Aβ concentration [270]. On the other hand, RAGE can mediate the Aβ reentry into the brain through BBB. Furthermore, at a nanomolar concentration, RAGE can bind with soluble Aβ [271], and this type of interaction is suggested in inflammatory, injuries, and AD brains [272,273]. Moreover, P-glycoprotein [274,275], megalin (i.e., gp330) and reticulon 4 receptor (RTN4R) [276,277] can play a role in Aβ trafficking, with their unknown contribution in Aβ transformation via BBB.

### 6.4. Altering the Interplay of APP, Aβ, and Tauopathy

In addition to the amyloid burden, as stated earlier, AD is closely associated with the accumulation of CTFs of the APP. Furthermore, AD is triggered by impaired APP metabolism and advances via tau pathology. Henceforth, blocking the impairment of APP metabolism is an effective way to reduce AD pathogenesis [278]. In an AD mouse model, it was observed that the production of BACE1 and Aβ, abnormal processing of APP can be reduced by mitochondria-targeted catalase [279]. Thus, to treat patients with AD, mitochondria-targeted molecules might be an effective therapeutic technique.

### 6.5. Altering the Interplay of Aβ and Neuroinflammation

Inflammation has a significant role in the pathology of AD [280]. Accumulation of Aβ and activation of PKR (i.e., a pro-apoptotic kinase) can take place due to the systemic inflammation. Henceforth, brain changes triggered by lipopolysaccharides are found to be largely mediated by PKR and might be a valid target to control the production of Aβ and neuroinflammation [281]. Moreover, in an APP/PS1/tau mouse model of AD, a current study has suggested that lixisenatide (i.e., glucagon-like peptide 1 receptor agonists) can reduce neuroinflammation, NFTs, and amyloid plaques [282]. As a result, lixisenatide might be developed as an effective novel treatment for AD [282].

## 7. Aβ Targeting Drugs

Over 50% of drug candidates in phase III clinical trials targeted Aβ as per the updated AD drug development pipeline in 2018. From 2017 to 2018, there was a sharp decline by 40% in anti-Aβ therapies in phase I and II clinical trials, which showed alteration in AD studies after the repetitive failures of anti-Aβ therapies [283] as shown in Table 1. Common mechanisms of anti-Aβ therapies include suppression of the Aβ plaque aggregation, decrease of Aβ_1-42_ production, or elevation of the clearance rate of Aβ from the brain and CSF [284]. Table 2 represents various ongoing clinical studies of anti-Aβ therapies for AD.

Indeed, the complex AD pathogenesis is still not clearly understood, which might include several biological mechanisms and many other proteins in addition to Aβ [307]. Perhaps this multifactorial nature of AD pathogenesis is the major cause for repetitive failures of anti-Aβ drugs since single target-based therapies might not be capable enough to tackle all the neurodegenerative event-related altered mechanisms [308].

## 8. Promising New Opportunities for Alzheimer’s Disease

Addressing the modifiable risk factors that play a role in AD development can be an alternative approach for therapeutics of AD [309]. Type 2 diabetes is one of such modifiable risk factors [310,311]. Neurons of the brain rely on the neurotrophic activities of insulin [312,313]. In the brain, resistance against the insulin’s neurotrophic properties can render neurons more susceptible to stress and also decrease the ability of the brain to repair the damages that accumulate over time, which can eventually cause deficits in metabolic, synaptic, and immune response activities [310]. Even in people without diabetes, insulin may lose its efficacy as a growth factor in the aged brain [314,315,316]. Thus, the use of antidiabetic agents may be considered as a useful technique in AD treatment [317]. In animal models of AD, liraglutide (i.e., an agonist of the glucagon-like peptide 1 receptor) has generated outstanding results [318]. Intranasal insulin also exerted impressive actions on cognition in individuals with AD or MCI in a small placebo-controlled study [319]. Multiple large controlled trials are also ongoing.

Neuroinflammation is another vital target for intervention. Multiple studies have revealed the link between AD and the immune system of the brain [320], where microglia plays a vital role in the association between neurodegeneration and inflammation [321]. On the other hand, epidemiological studies have revealed that a high lifetime burden of infectious diseases may elevate the risk of dementia or cognitive deficit in later life [322,323]. Peripheral inflammation and/or gut microbiota may cause activation of microglia and chronic activation may trigger harmful effects in certain situations. On the contrary, an effective adaptive immune system may avert the pathogenesis of AD via modulation of microglial activity [324]. However, further studies are required to understand microglia biology to design immune-based AD treatments.

Anti-tau therapies are also under investigation. Pathology of tau better correlates with cognitive deficits as compared to Aβ lesions. Therefore, once the clinical symptoms are apparent, targeting tau is likely to be more beneficial as compared to the clearance of Aβ. However, most of the early tau-targeting therapies were either based on the stabilization of microtubules or based on suppression of tau aggregation or kinases. Most of these therapies were discontinued due to their lack of efficacy and/or toxicity [325]. In recent times, most of the tau-based treatments in clinical studies are immunotherapies. Several preclinical studies have found them as promising [325].

There is a probability that unknown harmful neuronal stimuli can trigger compensatory changes during the early stage of the AD pathological process, which can eventually lead to decreased clearance or elevated generation of Aβ as a secondary result of the initial damage. One can draw parallels with sepsis is a life-threatening condition and can occur due to the dysregulation in the immune response. Sepsis initiates from an infectious agent, however, leukocytosis is a usual primary laboratory finding. Therapies are mainly aiming at the elimination of the main cause of the infection, rather than aiming at decreasing the numbers of white blood cells. Accumulation of Aβ may be regarded as a reaction of the brain in response to the neuronal damage. Therefore, treatments ought to have effects against the neuronal damage rather than the immune response of the host. Since initial leukocytosis in sepsis can trigger dysfunction in biological activities, thus the progressive and prolonged accumulation of Aβ in AD may have further toxic effects.

## 9. Conclusions

The amyloid cascade hypothesis was formulated based on strong histopathological, biochemical, and genetic evidence. Clinical, cognitive, and biomarker studies then further reinforced this hypothesis. Nevertheless, various classes of anti-Aβ agents that influence the production, aggregation, and clearance of Aβ have failed in clinical studies. Cognitive impairment was found to be accelerated by some agents that suppress the generation of nascent Aβ, including inhibitors of the γ-secretase and the BACE1. This aforesaid impairment may take place because of their off-target activities. Indeed, Aβ plays roles in memory consolidation and neuronal function. Increased generation of Aβ may take place in CNS under various clinical conditions, including cardiac arrest, general anesthesia, and sleep deprivation. Besides, Aβ generation can also be increased in response to neuronal stress. However, it is still not clear whether the accumulation of Aβ takes place due to the AD process or whether this accumulation is causing the disease. Current studies in asymptomatic or preclinical stages of AD and cognitively healthy people who are at risk of AD should provide some answers.

## Figures and Tables

**Table 1:** Major failed clinical trials of anti-Aβ therapeutics to treat Alzheimer’s disease.

Therapeutic Agent	Drug Class	Disease State of Participants	Clinical Trial Design	Reason of Failure	References
Avagacestat	γ-secretase inhibitor	Prodromal AD	Randomized, placebo-controlled phase II	No clinical efficacy; Adverse effects: Glycosuria, Weight loss	[285]
Atabecestat	BACE1 inhibitor	Early AD	Randomized, phase II/III	Adverse effects: Elevation of liver enzymes	[286,287,288]
Lanabecestat	BACE1 inhibitor	Mild-to-moderate AD	Randomized, phase III	Not likely to meet primary endpoint; terminated for futility	[289,290,291]
Verubecestat	BACE1 inhibitor	Mild-to-moderate AD	Randomized, placebo-controlled, double-blind phase III	No clinical efficacy; Adverse effects: rash, alterations in hair color, more chances of falls and injuries, weight loss, sleep disturbance, suicidal thoughts	[142,292]
Semagacestat	γ-secretase inhibitor	Probable AD	Double-blind, placebo-controlled phase III	No clinical efficacy, exacerbates cognitive functions at higher doses, increased incidence of skin cancer and infections	[142,155]
Tramiprosate	Aβ aggregation inhibitor	Mild-to-moderate AD	Phase III	No clinical efficacy	[173,293,294]
Gantenerumab	IgG1 humanized anti-Aβ mAbs	Prodromal AD	Randomized, placebo-controlled, double-blind phase III	Terminated owing to futility, no important differences were seen in primary and secondary endpoint	[295]
Crenezumab	IgG1 humanized anti-Aβ mAbs	Mild-to-moderate AD	Randomized, phase II	No clinical efficacy, Did not achieve primary and secondary endpoints.	[296]
Bapineuzumab	IgG1 humanized anti-Aβ mAbs	Mild-to-moderate AD	Placebo-controlled, double-blind phase III	No clinical efficacy	[297,298]

**Table 2:** Ongoing clinical studies of anti-Aβ therapeutics to treat Alzheimer’s disease.

Therapeutic Agent	Drug Class	Number of Participants	Disease State of Participants	Study Design	Clinical Trial Phase	Current Status	References
Aducanumab	IgG1 humanized anti-Aβ mAbs	2400	AD patients who had participated in previous studies 221AD103, 221AD301, 221AD302 and 221AD205	Open-label, multicenter trial	Phase III	Continuing	[299]
Gantenerumab and solanezumab	Monoclonal antibody	149	Early-onset AD caused by a genetic mutation	Randomized, placebo-controlled, double-blind trial	Phase II/III	Completed	[249]
BAN2401	Monoclonal antibody	1566	Early AD	Placebo-controlled, double-blind, parallel-group study	Phase III	Continuing	[263]
CAD106 CNP520	BACE inhibitor	480	Asymptomatic AD but carriers of homozygous APOE*ε4	Randomized, placebo-controlled, double-blind trial	Phase II/III	Continuing	[300]
Gantenerumab	Monoclonal antibody	1016	Early-onset AD	Multicenter, randomized, placebo-controlled, double-blind trial	Phase III	Continuing	[301,302]
Solanezumab	Monoclonal antibody	1150	Asymptomatic AD	Randomized trial	Phase III	Continuing	[303]
Sodium oligomannurarate (GV-971)	Aβ aggregation inhibitor	818	Mild to moderate AD	Randomized trial	Phase III	Completed	[304]
Gantenerumab	Monoclonal antibody	389	Mild AD	Randomized, placebo-controlled, double-blind trial	Phase III	Continuing	[305]
Albumin and Immunoglobulin	Polyclonal antibodies	347	Mild to moderate AD	Multicenter, randomized, controlled trial	Phase II/III	Completed	[306]
